# Prognostic models for short-term annual risk of severe complications and mortality in patients living with type 2 diabetes using a national medical claim database

**DOI:** 10.1186/s13098-023-01105-x

**Published:** 2023-06-15

**Authors:** Alexandre Vimont, Sophie Béliard, René Valéro, Henri Leleu, Isabelle Durand-Zaleski

**Affiliations:** 1 Assistance Publique Hôpitaux de Paris, URC-ECO, CRESS-UMR1153, Paris, France; 2 Public Health Expertise (PHE), Paris, France; 3 Department of Nutrition, Metabolic Diseases and Endocrinology, Aix Marseille University, APHM, INSERM, INRAE, University Hospital La Conception, Marseille, C2VN France

**Keywords:** Type 2 diabetes, Cardiovascular disease, Risk prediction, Health insurance claims

## Abstract

**Objective:**

Prognostic models in patients living with diabetes allow physicians to estimate individual risk based on medical records and biological results. Clinical risk factors are not always all available to evaluate these models so that they may be complemented with models from claims databases. The objective of this study was to develop, validate and compare models predicting the annual risk of severe complications and mortality in patients living with type 2 diabetes (T2D) from a national claims data.

**Research design and methods:**

Adult patients with T2D were identified in a national medical claims database through their history of treatments or hospitalizations. Prognostic models were developed using logistic regression (LR), random forest (RF) and neural network (NN) to predict annual risk of outcome: severe cardiovascular (CV) complications, other severe T2D-related complications, and all-cause mortality. Risk factors included demographics, comorbidities, the adjusted Diabetes Severity and Comorbidity Index (aDSCI) and diabetes medications. Model performance was assessed using discrimination (C-statistics), balanced accuracy, sensibility and specificity.

**Results:**

A total of 22,708 patients with T2D were identified, with mean age of 68 years and average duration of T2D of 9.7 years. Age, aDSCI, disease duration, diabetes medications and chronic cardiovascular disease were the most important predictors for all outcomes. Discrimination with C-statistic ranged from 0.715 to 0.786 for severe CV complications, from 0.670 to 0.847 for other severe complications and from 0.814 to 0.860 for all-cause mortality, with RF having consistently the highest discrimination.

**Conclusion:**

The proposed models reliably predict severe complications and mortality in patients with T2D, without requiring medical records or biological measures. These predictions could be used by payers to alert primary care providers and high-risk patients living with T2D.

**Supplementary Information:**

The online version contains supplementary material available at 10.1186/s13098-023-01105-x.

## Background

Type 2 diabetes (T2D) is one of the leading causes of morbidity and mortality worldwide, and the growing diabetes epidemic represents a major challenge to public health [1]. Informing both primary care physicians and patients at the individual level of the risk could allow better targeting of active prevention of complications.

Patients with T2D have a two- to threefold higher risk of suffering from a cardiovascular disease (CVD), including a higher risk of myocardial infarction (MI), stroke, unstable angina, and congestive heart failure and a higher rate of CVD-related death than the population not living with diabetes [2–4]. CV complications in diabetic patients are among the most prevalent complications [5], but other complications, such as metabolic disorder (ketoacidocetosis, severe hypoglycaemia etc.), acute renal failure, obliterating atherosclerosis of lower limbs or sepsis frequently occur, demonstrating a certain degree of severity of the disease [6].

Because of the chronicity and the progressive nature of the disease, long-term damage and organ failure may develop simultaneously or consecutively [7]. Predicting the onset of complications or mortality in the short term (annual risk) by physicians is more complicated than for long-term damage (≥ 5 years) but remains critical to ensure that appropriate preventive actions are taken.

Preventive medicine increasingly relies on modelling techniques to estimate the individual’s ‘absolute risk’ of a future event in order to inform therapeutic decisions. Risk models have been developed for patients living with diabetes, mostly using risk factors available from electronic medical records (e.g., body mass index, smoking status, biological and lipids markers, blood pressure) [8].

However, while these models are useful for clinical level risk prediction, they require information that is not always readily available without generalized electronic medical records and do not perform optimally when applied to populations other than the ones from which they were derived [8, 9].

Medical claims databases have emerged as an efficient source of data for disease monitoring surveillance [10]. Such databases collect automatically demographics and hospital diagnoses, procedures, medications generated through the provision of health services. With the growing use of patients’ personal insured accounts, payers have the ability to directly interact with primary care physicians and patients to communicate individual risks, so that general practitioners or specialists could identify high-risk profiles and recommend specific preventive actions. Reliable prediction of the short-term risk of acute events among patients with T2D based upon claims data would be an additional tool in the prevention of severe diabetes-related complications.

The objective of this study was to demonstrate the feasibility of developing prognostic models for short-term annual diabetes-related complications and mortality based on medical claims database and to compare the performance of different modelling approaches, including machine learning models.

## Method

### Data source

A representative sample of 1/97 of all insured individuals from the French National Health Data Information System (SNDS) which contains all medical claims for the entire French population was used [11]. This real-world database is managed by National Health Insurance (NHI) which ensures its representativeness from the general population based on sex, age, and location of residence [12]. In France, a compulsory public insurance scheme is applied to all individuals to cover the majority of costs. Information on private insurance schemes that patients subscribe to cover the complementary part was not available in the database. It includes information on demographics, medical history, diagnoses and procedures related to in-hospital admissions, prescriptions, laboratory assays, and date of death.

### Study design

A retrospective observational study design was used to model the risk of complications and mortality in patients with T2D (Additional file 1). The index date was set to the January 1st, 2014, corresponding to the inclusion date for all patients living with T2D to this date. Patients living with T2D were identified through a 2-year historical window before index date and were followed during a total of 4 years, to the December 31st, 2017, and censored after the first occurrence of a complication or death for the all-cause mortality analysis.

### Population

Patients living with T2D were included in the study and were identified in a 2-step process. Claims database may present some uncertainty for a reliable diagnostic based on ICD-10 between type 1 and type 2 diabetes, so that patients living with diabetes mellitus were first identified, and patients identified with type 1 diabetes criteria were excluded in a second step.

Adult patients living with diabetes mellitus were identified through a validated algorithm [13], based on 3 inclusion criteria evaluated during the 2-year historical window (2012 and 2013): individuals with ongoing long-term disease (LTD) with a diagnosis of diabetes mellitus (patients with at least 6-month treatment for diabetes are eligible for full reimbursement of healthcare costs capped at NHI tariff and the diagnosis is recorded in the claims database), individuals without LTD for diabetes but hospitalized with a primary or secondary diagnosis of diabetes mellitus, or individuals having 3 deliveries at different dates of oral antidiabetics or insulin, without LTD or diabetes-related hospitalization. Diagnoses codes were based on the ICD-10 classification and included sections E10.x, E11.x, E12.x, E13.x, E14.x.

Patients identified with type I diabetes were excluded from the study if they filled one of the following criteria: individuals with admissions for T1D, individuals under 45 years of age when insulin was first delivered, or individuals under 45 years of age at the start of LTD for diabetes. This advance age to identify type 1 diabetes was a conservative choice, but it limited the risk to incorrectly include patients with type I diabetes treated with insulin after 45 years, given that patients may not declared systematically LTD before this age, or that their entire history of healthcare consumption may not be available.

### Study outcomes

Study outcomes included severe CV complications, other severe complications, and all-cause mortality. Complications were considered severe because they were identified through diagnoses from recorded hospitalizations in the claims database. Complications managed solely in outpatient care could not be traced. Severe CV complications included admissions with primary diagnosis of heart failure (HF), peripheral arterial disease, myocardial infarction (MI), stroke, unstable angina (UA), transient ischemic attack (TIA) and CV-related death **(**Additional file 2**)**. Cardiovascular death was defined as death occurring at hospital during admission with a primary or secondary diagnosis of CV complications, or sudden cardiac arrest, cardiogenic shock, and other form of heart disorder. Other severe complications included admissions for metabolic disorder such as ketoacidosis coma, ketoacidosis, acidosis and hypoglycemia, acute renal insufficiency, amputation and sepsis of any type. All-cause mortality included in-hospital and out-hospital death occurring during the 4-year follow-up, regardless of the cause of death.

An additional analysis was conducted to assess ability of models to predict multiple complications within the same year (≥ 2 any complications separated by at least 30 days).

### Risk factors

For outcomes’ prediction, potential risk factors were derived from a 5-year historical window before index date. Risk factors included demographics (age, gender), diabetes-related information (long-term disease coverage for diabetes, duration of long-term disease, insulin and antidiabetic treatments), pre-existing chronic conditions (CVD, cardio-neurovascular disease, chronic respiratory disease, history of cancer or ongoing cancer, and inflammatory or rare disease), Charlson Comorbidity Index (CCI) [14], and adapted diabetes complications severity index (aDCSI) [15]. The aDCSI is a validated score used specifically with claims database and includes 7 categories of diabetes complications (ophthalmic, renal, neurologic, cerebrovascular, cardiovascular, peripheral vascular, and metabolic). Each category is scored with either 0 (no complication), 1 (non-severe complication), or 2 (severe complication), except for neurologic complications, which score a maximum of 1.

Diabetes medications (ATC class A01B) including sulfonylureas, meglitinides, metformin, thiazolidinediones, a-glucosidase inhibitors, GLP-1 receptor agonists, Dipeptidyl peptidase-4 inhibitors and insulin, were stratified and introduced into the models as follow: insulin only, insulin and antidiabetic treatment, antidiabetic monotherapy, bitherapy or tritherapy. Treatments for cardiovascular risk, such as b-blockers, calcium channel blockers, antihypertensive combinations were also included as covariates.

Pre-existing chronic conditions were identified through different medical algorithms combining LTD and history of chronic and acute diagnoses (Additional file 2). Overall, the presence of a condition was identified when individuals had ongoing LTD coverage for the condition, or individuals were hospitalized with a primary or secondary diagnosis related to the condition during the 5-year historical window before index date. The presence of LTD coverage and its duration were used as predictors.

Chronic cardiovascular disease, which was included in the CCI, was defined by chronic conditions diagnostic code (LTD) and recent acute complications including: coronary disease, stroke, chronic and acute heart failure, peripheral arterial disease, cardiac rhythm disorders and valvular disease occurring before the year preceding the index date (Additional file 2).

### Prognostic models

Pooling of repeated observations (PRO) was used to predict annual onset of each outcome. For each patient, the at-risk period was stratified into annual periods during which outcomes were observed. The PRO method pools observations over disjoint time intervals of equal length into a single sample to predict short-term risk of events [16]. Since risk factors vary over time, the method accounts for time dependent covariates, and risk factors were revaluated each year.

Distinct frameworks of prognostic models were developed, including standard approach with a logistic regression (LR) model, and machine learning approach with a random forest (RF) model and a neural network (NN) model. For LR model, the risk factors included in the final prognostic models were checked for multicollinearity and chosen using a stepwise variable selection approach based on Akaike’s Information Criterion with an expected p-value of 0.05.

The RF method was developed by Brieman [17] and relies on binary prediction trees. Variable selection is mechanically carried out because each split uses only a single covariate and approximates variable interaction through the hierarchical structure of the node splits. RF method corresponds to a collection of bootstrap samples that aim to reduce the variance of the prediction. For this study, RF used the SAS procedure HPFOREST, parametrized with a maximum number of 50 trees, a maximum of 20 nodes, a training fraction of 0.75 for each tree, and a leaf size of 10.

The NN model had a multilayer perceptron architecture, as described by Bishop [18], composed of several successive sets of neurons (layers). Parameters and weights were estimated by minimizing the loss function by using the SAS procedure HPNEURAL. The number of layers and neurons in each layer were fixed manually and step-by-step, by minimizing the average absolute error. An architecture of 4 layers was chosen, with k*4 neurons (being the number of risk factor, different in each set) for the first layer, k*2 for the second layer, k for the third layer and k/4 for the last layer.

### Measurement of performance

A k-fold cross-validation approach was used as an internal validation: the population was split into training sets (75%) and validation sets (25%). Training sets were used to develop the prognostic models, and validation sets were used to assess the performance of the models. The performance of the final prognostic models was evaluated based on discrimination, accuracy, sensitivity and specificity. The discrimination was assessed with the C-statistic that measures how well a prognostic model differentiates between patients with and without the outcome. It varies between 0.5 (no better than chance) and 1.0 (perfect discrimination) and corresponds to the proportion of subjects whose observed and predicted outcomes are concordant. Average C-statistics were reported based on a 100-fold cross-validation with replacement and 95% confidence intervals were estimated. P-values based on the Mann-Whitney U-statistics were calculated and the level of significance (1%) was adjusted for multiple comparisons (Bonferroni correction). Balanced accuracy was computed as a measure of overall predictive performance and the average of two proportions: the proportion correctly predicted for those who experienced the outcome (sensibility) and the proportion correctly predicted for those who did not (sensitivity). A score of 1 indicates a perfect model, and 0.5 indicates that the model is no better than chance. Balanced accuracy addresses the well-known phenomenon that binary classifiers tend to be biased toward the more frequent class, yielding an overly optimistic estimate of accuracy [19]. Sensitivity and specificity were determined using the threshold that maximized the sum of both.

Calibration of models were also presented as a goodness-of-fit. Calibration refers to the agreement between predicted and observed outcomes. Observed risk versus median predicted risk were compared for several sub-groups of patients. Calibration was also presented graphically with regression slopes of observed versus predicted risk for the different outcomes, and a 95% confidence interval calculated to evaluate the goodness of fit.

### Statistical analysis

These analyses were conducted in accordance with the TRIPOD (Transparent Reporting of a Multivariable Prediction Model for Individual Prognosis or Diagnosis) requirements [20]. Results are presented overall and for population subgroups (patients with and without chronic cardiovascular disease (CVD) and patients under and over 65 years of age). Sensitivity analyses were conducted by excluding HF in CV complications and by predicting multiple complications within the same year (≥ 2 any complications separated by at least 30 days). Additional analyses were conducted to assess robustness of models, by predicting the 1-year risk of outcomes during the 1st year only (without the PRO method), and by predicting the 4-year risk based on risk factors assessed at baseline only (without the PRO method). All analyses used SAS version 9.4 (SAS Institute Inc., Cary, NC, USA) on deidentified data with the approval of the French data protection authority (*Ref: MMS/MFI/AR1811775)* and informed consent was obtained from all subjects and/or their legal guardian(s).

## Results

### Population

A total of 25,549 patients were identified with diabetes mellitus, mostly identified through antidiabetic or insulin therapy (n = 21,339, 83.5%), ongoing long-term disease coverage for diabetes mellitus (n = 17,371, 68.8%),, or a recorded diagnosis of DM (n = 5,589, 21.8%) during the previous 2 years before the index date (Additional file 3). Among them, 2,541 patients (9.9%) identified with type 1 diabetes were excluded, and 22,708 patients with T2D were included in the study for analysis. Mean age was 68 years (11.4), 53% patients were male, and average duration of T2D was 9.7 years (7.5) (Additional file 4). 45% of patients had a CCI of 2 or more, in which T2D participated for 1 point. Predominant comorbidities were CVD (28%), cancer (12%) and chronic obstructive pulmonary disease (12%). For most patients, no previous severe diabetes-related complication was observed within the 5 previous years, as defined by the aDSCI, with 83% of patients having aDSCI evaluated to 0. A high proportion of patients with CVD was included, but most of them did not experience a recent CV complication (only 15% with aDSCI ≥ 1).

### Description of outcomes

The proportion of patients with any complication was 14,9% (n = 3,386) during the at-risk period (4 years), corresponding to an incidence rate of 41.4 severe complications per 1000 patients-year (Table 1**).** CV complications were predominant (77%), including HF (27%), peripheral arterial disease (13%), MI (7.5%), stroke (10%), UA (7%), TIA (7%) and CV-related death (5%). Other complications included metabolic disorder (7%), sepsis (7%), renal disorder (7%), and amputation (2%). Median time to events was 15 months [7–24] and no difference was observed between occurrence of CV complications and other complications. In average, similar proportions of complications were included between training (n = 2,874, 11.8%) and validation (n = 1,112, 13.4%) sets. The average 4-year mortality rates were 10.7% (n = 2,608) and 12.9% (n = 1,046) in the training and validation sets respectively.

**Table 1:** Incidence of severe complications during the 4-year risk period

Type of complication	Number (N, %)	Incidence per 1,000 pts-yr
**CV complications**	**2,606 (77%)**	**31.8**
Heart failure	924 (27.3%)	11.3
Peripheral arterial disease	429 (12.7%)	5.2
MI	253 (7.5%)	3.1
Stroke	349 (10.3%)	4.3
Unstable Angina	239 (7.1%)	2.9
TIA	226 (6.7%)	2.8
CV death	186 (5.5%)	2.3
**Other complications**	**780 (23%)**	**9.5**
Metabolic disorder	252 (7.4%)	3.1
Sepsis	234 (6.9%)	2.9
Renal disorder	241 (7.1%)	2.9
Amputation	53 (1.6%)	0.6
**All complications**	**3,386 (100%)**	**41.3**

Note: CV, Cardiovascular; MI, Myocardial Infarction; pts-yr: Patient-year;

TIA Transient Ischemic Attack

### Comparison of patients with and without severe complication

Patients with any complication during the complete at-risk period were older (median age: 74 vs. 67 years old), had a higher CCI (index > 1: 68% vs. 41%) and a higher aDCSI (score > 0: 37% vs. 14%) at baseline. Without age adjustment, they were more likely to have chronic cardiovascular disease (53% vs. 23%), cancer (15% vs. 12%), COPD (17% vs. 11%), neurologic disease (6% vs. 4%), and chronic kidney failure (2% vs. 0.5%).

### Models’ performance

Overall, models performed well in predicting severe diabetes-related complications and all-cause mortality. with C-statistic being superior to 0.70 in average for all models in validation datasets and RF outperforming other models for accuracy and discrimination (Table 2) although not being statistically superior on all outcomes.

**Table 2:** Comparative performance for severe complications risk prediction models

	Training sets C-statistic	Validation sets						
		C-statistic	95%CI	p-value:		Se	Sp	Balance accuracy
				**vs. LR**	vs. RF			
**CV complications**								
LR	0.738	**0.715**	0.658–0.772	Ref	0.1271	73%	60%	**0.665**
RF	0.944	**0.786**	0.747–0.825	0.1271	Ref	71%	72%	**0.717**
NN	0.739	**0.738**	0.686–0.79	0.3317	0.1787	70%	67%	**0.685**
**Other complications**								
LR	0.738	**0.706**	0.653–0.759	Ref	< 0.0001	59%	70%	**0.645**
RF	0.981	**0.847**	0.800-0.894	< 0.0001	Ref	80%	81%	**0.801**
NN	0.732	**0.670**	0.626–0.714	0.2279	< 0.0001	74%	52%	**0.630**
**All-cause mortality**								
LR	0.823	**0.814**	0.769–0.859	Ref	0.0776	76%	73%	**0.745**
RF	0.941	**0.860**	0.821–0.899	0.0776	Ref	80%	79%	**0.794**
NN	0.845	**0.841**	0.791–0.891	0.2354	0.3486	82%	71%	**0.765**

Note: CV, Cardiovascular; LR, Logistic Regression; NN, Neural Network; RF, Random Forest

Balanced accuracy ranged from 0.665 to 0.717 for CV complications, from 0.645 to 0.801 for all complications and from 0.745 to 0.794 for all-cause mortality, suggesting models are more accurate at predicting severe complications and mortality than chance alone, with RF having systematically the highest accuracy. Discrimination with C-statistics ranged from 0.715 to 0.786 for CV complications, from 0.670 to 0.847 for all complications and from 0.789 to 0.837 for all-cause mortality, with NN having consistently the highest discrimination.

Subgroup analyses and sensitivity analyses are presented in Table 3. Discrimination was systematically superior in patients without CVD, and in patients under 65 years of age. Additional sensitivity analyses suggested similar performances when predicting outcomes during the first year of follow-up only, or when predicting outcomes within the 4-year period from baseline risk factors (without PRO method) (Table 3). Results were shown to be robust in predicting 2 or more events within one year, with slightly better performance than predicting a single event.

**Table 3:** Comparative performance for subgroup and sensitivity analyses (C-statistic)

C-statistic		Subgroup-analyses				Sensitivity analyses					
	Reference	Without CVD	With CVD	< 65 years	≥ 65 year	Excluding HF		Multiple event		1st year only	4-year risk
**CV complications**											
LR	**0.715**	0.679	0.642	0.811	0.669	0.753		0.752		0.701	0.688
RF	**0.786**	0.739	0.722	0.848	0.788	0.796		0.839		0.793	0.769
NN	**0.738**	0.693	0.647	0.84	0.699	0.785		0.78		0.716	0.724
**Other complications**											
LR	**0.706**	0.664	0.676	0.776	0.668	0.737		0.731		0.705	0.691
RF	**0.847**	0.809	0.789	0.949	0.797	0.869		0.885		0.857	0.837
NN	**0.670**	0.619	0.609	0.748	0.649	0.727		0.684		0.654	0.661
**All-cause mortality**											
LR	**0.814**	0.793	0.761	0.877	0.762	-		-		0.814	0.793
RF	**0.860**	0.844	0.795	0.931	0.823	-		-		0.860	0.855
NN	**0.841**	0.833	0.756	0.928	0.787	-		-		0.841	0.835

Note: CV, Cardiovascular; HF, Heart Failure; LR, Logistic Regression; NN, Neural Network; RF, Random Forest

Including the type of resource used during the last year (number of visits at general practitioner, ophthalmologist, cardiologist, endocrinologist, dental and other specialist, and number of biological assays for HbA1c, glycaemia, creatinine, albuminuria and lipids, and ECG, number of admissions, number of days, outpatient costs) (not shown) was assessed but did not improve model performance.

### Risk factors

The same set of predictors were introduced in all models across outcomes. Age was the risk factor with the largest impact on the risk of having a severe complication, whether with LR or RF (Additional file 5), followed by the aDSCI. Insulin and antidiabetic medications were associated to an increased and protective effect respectively, reflecting a selection bias for which more severe patients are prescribed insulin. Treatments for cardiovascular risk was not retained by the LR model but was in the top 5 of risk factors that contributed the most for the RF model. Other risk factors, such as CVD, psychiatric disorders and chronic end-stage renal disease were consistently associated with a significant risk of event across outcomes. Additionally, for all-cause mortality, COPD, liver or pancreas disease, cancer and neurodegenerative disease were associated to an increased risk of death, whether with LR or RF.

### Calibration and predicted risks

Calibrations of models were presented for several subgroup populations (Additional file 7).

Overall, all models underestimated risk of outcomes, particularly for patients with severe profiles and for all-cause mortality.

Patients with observed severe complications were at 2 to 3-fold increased predicted risk of any complication, and patient with observed death were at 5 to 8-fold increased risk of all-cause mortality. Patients with pre-existing CVD at baseline were at 3 to 4-fold increased risk of any complication and of all-cause mortality. Patient over 75 years of age at baseline were at 2 to 3-fold increased risk of CV complication and 3 to 6-fold increased risk of all-cause mortality. Similarly, patients without treatment or with insulin therapy, alone or in association, were at 2 to 4-fold increased risk of any complications or all-cause mortality.

**Fig. 1:**
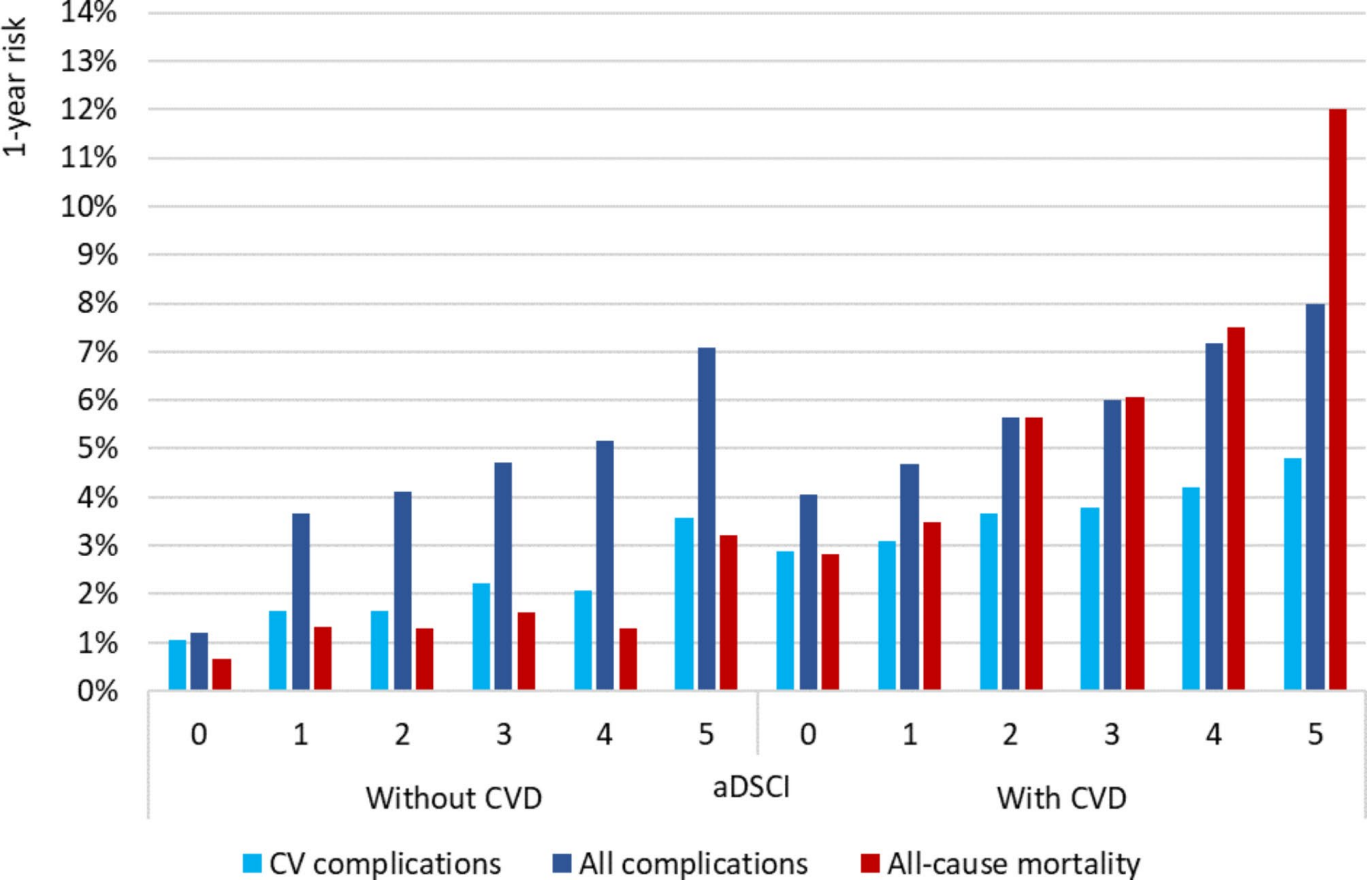
Annual median predicted risks by Random Forest stratified by aDSCI and prior CVD

One-year risks stratified by aDSCI and CVD were also presented for the RF model (Fig. 1). In patients with CVD, risks increased with aDSCI score, reaching 12% of death rate for patient with an aDSCI of 5. In patients without CVD, risks of CV complication or all-cause mortality were similar between patients with aDSCI between 0 and 4, while the risk of any complication increased with aDSCI.

## Discussion

We developed prognostic models for the annual risk of severe complications in patient living with T2D, including CV complications, other severe complications and all-cause mortality. The originality of this study lies in the fact that we used exclusively data from a national claims database and it compared different modelling approach, including logistic regression model and machine learning approach with random forest and neural network. We demonstrated the feasibility of applying these approaches to medical claims data to predict the annual risk of complication in this population.

The main finding of this study was that RF model performed well, although not being statistically superior to other models, in predicting severe complications and all-cause mortality with simple and few risk factors derived from medical claims database, such as age, LTD, antidiabetic medications, aDSCI, history of CVD and presence other comorbidities. This framework makes possible a routine evaluation of the different individual risks by the national health insurance communicated for patients and for primary care providers to identify high-risk patients and to personalize prevention.

In a recent meta-analysis on CVD prediction in patients with T2D, authors reported that C-statistics ranged from 0.64 to 0.80 for prognostic models developed in patient living with diabetes [8]. In their meta-analysis, authors estimated a pooled C-statistics of 0.67 based on validation studies of the following prognostic models: UKPDS calculator [21], the ADVANCE model [22], the DCS model [23], the Fremantle model [24] and the NDR model [25]. Our study highlights results from LR, RF and NN models were comparable with the existing findings for CV prognosis [26].

The crucial difference between models developed in this study and models included in this meta-analysis lies in the available risk factors, which were derived from biological results and medical records in the latter. In contrast, we used only information that is available from a claims database. Our study demonstrated the feasibility of prognostic models based on claims information and the possibility to derive relevant risk factors such as diabetes duration (available with LTD duration), history of diabetes-related complications (aDSCI) and antidiabetic treatments which are not systematically included in all clinical models described above. However, some risk factors identified in the UKPDS, NDR, or Advance models remain difficult to identify, and thus, limit the predictive accuracy. For example, smoking or obesity do exist, but are typically underreported in medical claims.

Prognostic models did consider diabetes therapeutic strategies during the study period. Adjustment was made for patients without treatment, or patients receiving insulin and/or antidiabetic medication. This analysis conducted with LR showed that patients receiving antidiabetic treatments were at lower risk of outcome (CV or other complications and all-cause mortality) compared to patients receiving no treatment.

Concerning CV outcomes, the comparison with the literature may be limited as existing studies mostly predict composite of strong endpoints (MI, stroke, CVD-related death, and CHD). In our study, we enlarged CV outcomes to other severe DM-related complications (UA, TIA and peripheral arterial disease) because they were also associated to major morbidity in DM patients. Hospitalization for CHF represented an important part of CV complications (~ 25%) in our study. Excluding CHF in CV complications was associated to a deterioration in discrimination, with C-statistics decreasing from 2 to 4 points.

To our knowledge, this study is also the first study to include a wide range of complications requiring hospitalization such as metabolic disorder with ketoacidosis coma, ketoacidosis, acidosis, hypoglycaemia, sepsis, acute renal insufficiency, and amputations. Model performances for predicting other complications were similar to those for CV complications, with better performance for RF model.

Machine learning models performed better than logistic regression to predict mortality and had better performance than the existing literature. In a recent study predicting the 5-year mortality risk in older adults with T2D [27], the authors presented balance accuracy of 0.77 and C-statistics of 0.74 with model including key risk factors, such as biological markers, BMI, and smoking. Our study showed that without the latest risk factors, but with other key risk factors (disease duration, aDSCI and diabetes medications), similar results can be achieved.

A second finding was that RF had the best accuracy and discrimination among models. Compared to a similar claims database study that predicted the 3-year risk of adverse outcomes with machine learning, discrimination performance of RF (C-statistics 0.77–0.86) model in our study was relatively close to the performance with complex machine learning models (C-statistics = 0.77), such as gradient boosting decision tree, recurrent neural networks, multilayer perceptron and transformers [28, 29]. On a broader perspective, machine learning models have been shown to be replicable and transferrable to local healthcare systems when built on a national database, while a model constructed on a local level cohort was difficult to transfer to a national level [30]. On top, factors that usually contribute to risk of bias, including small study size, poor handling of missing data, and failure to deal with overfitting were not present in this study [31].

Also, this study was conducted on a sample of the national insured population, whose representativeness was ensured by a statistical process conducted by the NHI based on the precise repartition of sex, age, and location of residence. This methodology limited the potential underrepresentation of sub-group of patients with type 2 diabetes and support the generalizability of our results. The nature of predictors, that are essentially based on admissions and diabetes treatments, and the transparent parametrizations of RF and NN support also the generalizability of the results.

Survival models based on hazard ratio estimation were not included in our framework for two reasons: first, they were considered to use a different approach with instantaneous risk estimation which is different than binary outcome prediction, and second, dates were only available on a monthly basis, for anonymous purposes, which would have certainly underestimated performance of these models. Additionally, formal sensitivity analyses to assess authors choices for RF and NN parametrizations were not presented since results were stables over the different parametrizations.

Main predictors identified with LR and RF were age and history of diabetes related complications described with aDSCI, diabetic medications, and diabetes duration which was consistent with the literature [32, 33]. Additionally, chronic CV disease, psychiatric disorder and chronic end-stage renal disease were consistently associated with a significant risk of event across outcomes. This study also confirmed that the aDSCI, initially developed for the prediction of mortality and risk of hospitalization [34], was an important predictor for complications and all-cause mortality from this claims database.

The first limitation was the identification of study outcomes, based on probabilistic algorithms using health insurance claims data that have not been fully validated, which could lead to the misclassification of outcomes and comorbidities. Severe complications may be underestimated since events occurring in outpatient settings could not be gathered, so that models assumed that most of severe complications were treated at hospital. Second, patients may have experienced complications or major events prior to the start of data availability, such as CVD, and comorbidities may have been underreported or misclassified. Third, a recorded diagnostic code on a medical claim may be inaccurate. Taking that into account, authors applied additional inclusion criteria to differentiate type 1 and type 2 diabetes by using a minimum onset age of 45 years, conjointly applied with insulin delivery or LTD, before which patients were assumed to have type 1 diabetes. Despite the criteria used to differentiate type 1 and type 2 diabetes, potential misclassification of diabetes type remains a limitation.

Finally, censoring patients at 1st occurrence of event prevented the prediction of multiple and recurrent complications. However, our sensitivity analyses showed robustness in predicting 2 or more events within one year, with slightly better performance than predicting a single event.

Risk evaluation is essential to individualize therapy and is encouraged by clinical practice guidelines for the management of risk factors. However, in practice, biological risk factors are not all systematically available and health professionals other than diabetologist may not be familiar with clinical models, which can limit their use in some cases. The use of routine prediction models from real-world claims database is to provide a transparent platform to communicate this risk to the patient and help all health professionals to make a quick and accurate assessment of their patients’ risk and optimize their management care.

Medical claims databases are a valuable resource to develop prognostic models that have a strong potential to identify patients at high risk of complications within a certain time window [35, 36]. Risks could be routinely assessed by the national health insurance, owner of the data, and communicate thereafter to patients, and primary care providers to personalize prevention.

## Conclusion

Models based on national medical claims data could reliably predict severe complications and mortality in patients with T2D, without requiring medical records or biological measures. These models could be relevant for all health professionals to identify high-risk patients and optimize their monitoring, and more generally for payers to implement preventive measures.

## Electronic supplementary material

Below is the link to the electronic supplementary material.

Supplementary Material 1

## Data Availability

The datasets analyzed during the current study are not publicly available due to the mandatory approval and authorization needed from the French regulatory institution. Aggregated data are available from the corresponding author on reasonable request.

## List of Abbreviations

aDSCI: Adapted Diabetes Severity and Comorbidity index
ATC: Anatomical Therapeutic Chemical
BMI: Body Mass Index
CCI: Comorbidity Charlson Index
CHF: Chronic Heart Failure
COPD: Chronic Obstructive Pulmonary Disease
CV: Cardiovascular
CVD: Cardiovascular Diseasese
DM: Diabtes Mellitus
LR: Logistic Regression
LTD: Long-Term Disease
MI: Myocardial Infarction
NDR: Swedish National Diabetes Register
NHI: National Health Insurance
NN: Neural Network
PRO: Pooling of repeated observations
RF: Random Forest
T2D: Type 2 diabetes
TIA: Transient Ischemic Attack
UA: Unstable Angina
