# Supplementary material for: Prognostic models for short-term annual risk of severe complications and mortality in patients living with type 2 diabetes using a national medical claim database
Source: Diabetol Metab Syndr. 2023 Jun 15;15:128. doi: 10.1186/s13098-023-01105-x (PMC10268447; doi:10.1186/s13098-023-01105-x)
Supplement: Supplementary file 1 — Supplementary Material 1 [file 13098_2023_1105_MOESM1_ESM.docx]

Supplementary Information

Additional file 1. Study design

Start of inclusion

(01/01/2012)

Start of study

(01/01/2009)

Identification period for comorbidities

[5 years]

Index date

(01/01/2014)

At risk period for

study outcomes
[4 years]

End of follow-up period

(31/12/2017)

Inclusion period for individuals with DM

[2 years]

Additional file 2. Definition of outcomes and risk factors.

**Table 1 Definition of severe acute complications**

| **Type of complication** | **ICD-10-CM*** |
| --- | --- |
| Myocardial infarction | I21.x Acute Myocardial infarction (MI)  I22.x Repetition of MI  I23.x Certain current complications following ST elevation (STEMI) and non-ST elevation (NSTEMI) myocardial infarction  I24.x Other Acute heart disease |
| Stroke | I63.x Cerebral Infarction  I64.x Stroke, unspecified |
| Unstable angina | I20.0 Unstable angina pectoris |
| Transient ischemic attack | G45 Transient ischemic attack  I65.x Occlusion and stenosis of precerebral arteries, not leading to a cerebral infarction  I66.x Cerebral artery occlusion and stenosis, not leading to a cerebral infarction |
| Peripheral arterial disease | I74.x Arterial embolism and thrombosis  I70.2 Atherosclerosis of the distal arteries  K55.0 Acute vascular disorders of the intestine  N28.0 Kidney ischemia and infarction  I63.0-I63.2 Cerebral Infarction due to thrombosis or embolism |
| CV-related death | Death occurred during hospitalisation associated to an acute cardiovascular event (above) OR one of the following other CV events (below): |
| Sudden cardiac arrest | I46 Sudden cardiac arrest |
| Cardiogenic shock | R57.x Cardiogenic shock |
| Other cerebrovascular events | I67.x Other cerebrovascular diseases  I68.x Other Cerebrovascular disorders |
| Other cardiovascular events | I27.x Other cardiopulmonary disorder  I30.x-I49.x Other forms of heart disease  I51.x Complications of heart disease  I52.x Other heart disease |
| Other acute complications | *Metabolic disorder*  Acidocetosic Coma: E10.0, E11.0, E12.0, E13.0, E14.0  Ketoacidosis E10.1, E11.1, E12.1, E13.1, E14.1  E87.0 Hyperosmolarities and hypernatremias, unspecified  E87.2 Acidosis  E16.2 Hypoglycemia, unspecified  *Renal disorder*  N17.x Acute renal insufficiency  *Amputation*  R02 Gangrene  S88.9 Traumatic leg amputation, level unspecified  S90.x Superficial traumatic injury of the ankle and foot  S91.x Open wound of the ankle and foot  S98.x Traumatic ankle and foot amputation  M86.x7 Osteomyelitis, ankle and foot  *Sepsis*  A39.2 Meningococcal sepsis  A40.x Streptococcal sepsis  A41.x Other sepsis  R57.2 Septic shock  R65.x Systemic Inflammatory Response Syndrome |

*Roots of ICD-10 only are presented, but all sub-class of ICD-10 categories are included.

**Table 2 Definition of comorbidities**

| **Comorbidity** | **Identification in the database** |
| --- | --- |
| Chronic cardiovascular disease | - Individuals with ongoing Long-term disease (LTD) covering in year *n* associated to ICD-10 codes below - AND/OR individuals hospitalized during at least one of the last 5 years (n-1 to n-4) for these same reasons with primary diagnosis (MD) or related diagnosis (RD) - AND/OR individuals hospitalized in year *n* for any other reason but with any ICD-10 codes below as an associated complication or morbidity (AD) - AND without hospitalization in year *n* for the same ICD-10 codes below. |
| Chronic coronary disease | I20.x Unstable angina pectoris  I21.x Acute myocardial infarction  I22.x Repetition of MI  I23.x Certain current complications following ST elevation (STEMI) and non-ST elevation (NSTEMI) myocardial infarction  I24.x Other Acute heart disease  I25.x Chronic ischemic heart disease |
| Stroke sequalae | I60.x Subarachnoid hemorrhage  I61 .x Intracerebral hemorrhage  I62.x Other non-traumatic intracranial hemorrhage  I63.x Cerebral infarction  I64.x Stroke, not specified as hemorrhagic or infarct  I67.x Other cerebrovascular disease  I68.x Cerebrovascular disorders during diseases classified elsewhere  I69.x Sequelae of cerebrovascular disease |
| Chronic heart failure | I50.x Heart failure  I11.0 Hypertensive heart disease, with congestive heart failure  I13.0 Hypertensive cardio-nephropathy, with heart failure  I13.2 Hypertensive cardio-nephropathy, with heart congestive failure  I13.9 Hypertensive cardio-nephropathy, unspecified |
| Peripheral arterial disease | I70.2 Atherosclerosis of distal arteries  I73.9 Peripheral vascular disease, unspecified  I74.0 Embolism and thrombosis of the abdominal aorta  I74.3 Embolism and thrombosis of the arteries of the lower limbs  I74.4 Embolism and thrombosis of distal arteries, unspecified  I74.5 Embolism and thrombosis of the iliac artery |
| Cardiac rhythm disorders | I44.x Left bundle branch and atrioventricular block  I45.x Other conduction disorders  I47.x Paroxysmal tachycardia  I48.x Atrial fibrillation and flutter  I49.x Other cardiac arrhythmias |
| Valvular disease | I05.x Rheumatic diseases of the mitral valve  I06.x Rheumatic diseases of the aortic valve  I07.x Rheumatic diseases of the tricuspid valve  I08.x Multiple valve diseases (rheumatic)  I34.x Non-rheumatic mitral valve diseases  I35.x Non-rheumatic attacks of the aortic valve  I36.x Non-rheumatic attacks of the tricuspid valve  I37.x Non-rheumatic pulmonary valve diseases  I38.x Endocarditis, valve not specified  I39.x Endocarditis and heart valve involvement during |
| Cancer | - Individuals with ongoing LTD in year *n* associated to ICD-10 codes below - AND/OR individuals hospitalized during at least one of the last 5 years (n-1 to n-4) for these same reasons (MD or RD)   Breast cancer (C50, D05)  Colorectal cancer (C18, C19, C20, D01.0, D01.1, D01.2)  Lung cancer (C33, C34, D02.1, D02.2)  Prostate cancer (C61, D07.5)  Other cancer (CXX.x) |
| Psychiatric condition | - Individuals with ongoing LTD in year *n* associated to ICD-10 codes below - AND/OR individuals hospitalized during at least one of the last 2 years (n-1 to n-4) for these same reasons (MD or RD)   Psychotic disorder (F20-F25, F28, F29)  Neurotic and mood disorders (F30-F34, F38-F45, F48)  Mental disability (F70-F73, F78, F79)  Addictive disorders (F10-F19) |
| Neurologic | - Individuals with ongoing LTD in year *n* associated to ICD-10 codes below - AND/OR individuals hospitalized during at least one of the last 5 years (n-1 to n-4) for these same reasons (MD or RD)   multiple sclerosis (G35), paraplegia (G82), myopathy or myasthenia (G70-G73), epilepsy (G40-G41), dementia (including Alzheimer's disease), Parkinson's disease |
| COPD | - Individuals with ongoing LTD in year *n* associated to ICD-10 codes below - AND/OR individuals hospitalized during at least one of the last 5 years (n-1 to n-4) for these same reasons (MD or RD)   Chronic obstructive pulmonary disease (J40-J47) |
| Inflammatory or rare disease | - Individuals with ongoing LTD in year *n* associated to ICD-10 codes below - AND/OR individuals hospitalized during at least one of the last 5 years (n-1 to n-4) for these same reasons (MD or RD)   Chronic inflammatory bowel disease (K50-K51, M07.4, M07.5)  Rheumatoid arthritis or related diseases (M05, M06, M08, M09)  Other (M30-M36)  Cystic fibrosis (E84)  HIV (B20-B24) |
| Chronic kidney failure | At least 45 days treatment with hemodialysis  Renal transplantation |
| Liver or pancreas disease | - Individuals with ongoing LTD in year *n* associated to ICD-10 codes below - AND/OR individuals hospitalized during at least one of the last 5 years (n-1 to n-4) for these same reasons (MD or RD)   B18, I85, K70-K76, K85, K86 |

Additional file 3 Patient flowchart

Included patients

with type 2 diabetes

N =22,708

Excluded patients

with type 1 diabetes

N =2,541

Individuals with ongoing long-term allowance for DM

N = 17,371 (68.8%)

Included patients with diabetes mellitus

N =25,549

Validation set

N = 5,677 (25%)

Complications: n=855

Deaths: n=644

Training set

N = 17,031 (75%)

Complications: n=2,531

Deaths: n=1,933

Individuals with recorded diagnoses (main, related or associated)

during the years 2012 or 2013

N=5,589 (21.8%)

Individuals who received at least 3 deliveries (on different dates) of oral antidiabetics or insulin
during the years 2012 or 2013

N = 21,339 (83.5%)

Additional file 4. Comparison of risk factors among patients with and without complication during the at-risk period

| Variable | | | No complication  (N=19,322) | | Complication  (N=3,386) | | Total  (N=22,708) |
| --- | --- | --- | --- | --- | --- | --- | --- |
| Male (n, %) | | | 9974 (51.6%) | | 1980 (58.5%) | | 11954 (52.6%) |
| Age, year (mean, sd) | | | 67.1 (11.2) | | 72.8 (10.9) | | 68.0 (11.4) |
| Median (Q1-Q3) | | | 67 (59 – 76) | | 74 (65 – 81) | | 67 (60-77) |
| Age group (n, %) | | |  | |  | |  |
| 35-54 | | | 2665 (13.8%) | | 191 (5.6%) | | 2856 (12.6%) |
| 55-64 | | | 5480 (28.4%) | | 619 (18.3%) | | 6099 (26.9%) |
| 65-74 | | | 5870 (30.4%) | | 920 (27.2%) | | 6790 (29.9%) |
| 75et+ | | | 5307 (27.5%) | | 1656 (48.9%) | | 6963 (30.7%) |
| LTD for diabetes (n, %) | | | 13209 (68.4%) | | 2064 (61.0%) | | 15273 (67.3%) |
| Duration | | | 9.2 (7.2) | | 12.8 (8.7) | | 9.7 (7.5) |
| Antidiabetic treatment strategies | | |  | |  | |  |
| None | | | 1052 (5.4%) | | 205 (6.1%) | | 1257 (5.5%) |
| Insulin only | | | 1156 (6.0%) | | 473 (14.0%) | | 1629 (7.2%) |
| Insulin + antidiabetic | | | 2349 (12.2%) | | 687 (20.3%) | | 3036 (13.4%) |
| Antidiabetic only | | | 14765 (76.4%) | | 2021 (59.7%) | | 16786 (73.9%) |
| Bitherapy | | | 6180 (32.0%) | | 870 (25.7%) | | 7050 (31.0%) |
| Monotherapy | | | 7309 (37.8%) | | 950 (28.1%) | | 8259 (36.4%) |
| Tritherapy | | | 1276 (6.6%) | | 201 (5.9%) | | 1477 (6.5%) |
| Antidiabetic treatments | | |  | |  | |  |
| Biguanide | | | 12176 (63.0%) | | 1712 (50.6%) | | 13888 (61.2%) |
| Sulfonamide | | | 7077 (36.6%) | | 1192 (35.2%) | | 8269 (36.4%) |
| Dipeptidyl peptidase-4 inhibitors (DPP4) | | | 2942 (15.2%) | | 518 (15.3%) | | 3460 (15.2%) |
| Alpha-glucosidase inhibitors (AGI) | | | 783 (4.1%) | | 147 (4.3%) | | 930 (4.1%) |
| GLP-1 receptor agonists (GLP-1-RA) | | | 743 (3.8%) | | 122 (3.6%) | | 865 (3.8%) |
| Other | | | 1881 (9.7%) | | 460 (13.6%) | | 2341 (10.3%) |
| Cardiovascular treatments | | |  | |  | |  |
| Angiotensin-converting-enzyme inhibitors (ACE) | | | 11154 (57.7%) | | 2266 (66.9%) | | 13420 (59.1%) |
| Antithrombotic | | | 7934 (41.1%) | | 2243 (66.2%) | | 10177 (44.8%) |
| Beta-blockers | | | 5800 (30.0%) | | 1533 (45.3%) | | 7333 (32.3%) |
| Calcium channel blockers (CCB) | | | 3914 (20.3%) | | 1027 (30.3%) | | 4941 (21.8%) |
| Diuretics | | | 3233 (16.7%) | | 1277 (37.7%) | | 4510 (19.9%) |
| Antihypertensives | | | 1174 (6.1%) | | 362 (10.7%) | | 1536 (6.8%) |
| Comorbidity Charlson Index (CCI) (n, %) | | |  | |  | |  |
| 1 | | | 11316 (58.6%) | | 1101 (32.5%) | | 12417 (54.7%) |
| 2 | | | 2459 (12.7%) | | 479 (14.1%) | | 2938 (12.9%) |
| 3 | | | 3313 (17.1%) | | 908 (26.8%) | | 4221 (18.6%) |
| >3 | | | 2234 (11.6%) | | 898 (26.5%) | | 3132 (13.8%) |
|  |  |  | |  | |  |  |
| aDSCI |  |  | |  | |  |  |
| 0 | | | 16663 (86.2%) | | 2138 (63.1%) | | 18801 (82.8%) |
| 1 | | | 1185 (6.1%) | | 366 (10.8%) | | 1551 (6.8%) |
| 2 | | | 891 (4.6%) | | 386 (11.4%) | | 1277 (5.6%) |
| 3 | | | 211 (1.1%) | | 143 (4.2%) | | 354 (1.6%) |
| 4 | | | 187 (1.0%) | | 134 (4.0%) | | 321 (1.4%) |
| ≥5 | | | 185 (1.0%) | | 219 (6.5%) | | 404 (1.8%) |
| Comorbidities (n, %) | | |  | |  | |  |
| Chronic cardiovascular disease | | | 4483 (23.2%) | | 1786 (52.7%) | | 6269 (27.6%) |
| Coronary disease | | | 2221 (11.5%) | | 910 (26.9%) | | 3131 (13.8%) |
| Stroke | | | 674 (3.5%) | | 228 (6.7%) | | 902 (4.0%) |
| Chronic heart failure | | | 546 (2.8%) | | 369 (10.9%) | | 915 (4.0%) |
| Peripheral arterial disease | | | 739 (3.8%) | | 498 (14.7%) | | 1237 (5.4%) |
| Cardiac rhythm disorders | | | 1216 (6.3%) | | 613 (18.1%) | | 1829 (8.1%) |
| Valvular disease | | | 259 (1.3%) | | 157 (4.6%) | | 416 (1.8%) |
| Other | | | 207 (1.1%) | | 84 (2.5%) | | 291 (1.3%) |
| Cancer | | | 2250 (11.6%) | | 498 (14.7%) | | 2748 (12.1%) |
| Psychiatric condition | | | 1162 (6.0%) | | 218 (6.4%) | | 1380 (6.1%) |
| Neurologic | | | 923 (4.8%) | | 220 (6.5%) | | 1143 (5.0%) |
| COPD | | | 2136 (11.1%) | | 564 (16.7%) | | 2700 (11.9%) |
| Inflammatory or rare disease | | | 569 (2.9%) | | 110 (3.2%) | | 679 (3.0%) |
| Chronic kidney failure | | | 84 (0.4%) | | 78 (2.3%) | | 162 (0.7%) |
| Liver or pancreas disease | | | 630 (3.3%) | | 150 (4.4%) | | 780 (3.4%) |
| Healthcare resources during the year before baseline | | | | |  | |  |
| Biological exams | | |  | |  | |  |
| ≥ 2 HBA1c controls (n, %) | | | 14278 (73.9%) | | 2522 (74.5%) | | 16800 (74.0%) |
| ≥ 2 glycaemia control (n, %) | | | 10186 (52.7%) | | 1890 (55.8%) | | 12076 (53.2%) |
| ≥ 2 creatinine control (n, %) | | | 12631 (65.4%) | | 2298 (67.9%) | | 14929 (65.7%) |
| ≥ 2 lipid control (n, %) | | | 668 (3.5%) | | 140 (4.1%) | | 808 (3.6%) |
| ≥ 1 ECG control (n, %) | | | 5244 (27.1%) | | 1337 (39.5%) | | 6581 (29.0%) |
| Total biological exams(mean, sd) | | | 2.0 (3.1) | | 2.4 (4.6) | | 2.1 (3.4) |
| Health professionals visits | | |  | |  | |  |
| ≥ 1 endocrinologist visit (n, %) | | | 2504 (13.0%) | | 498 (14.7%) | | 3002 (13.2%) |
| ≥ 1 cardiologist visit (n, %) | | | 4767 (24.7%) | | 1261 (37.2%) | | 6028 (26.5%) |
| ≥ 1 ophthalmologist visit (n, %) | | | 5408 (28.0%) | | 947 (28.0%) | | 6355 (28.0%) |
| GP visits (mean, sd) | | | 7.8 (6.1) | | 9.8 (7.5) | | 8.1 (6.4) |
| Nurse visits (mean, sd) | | | 31.7 (99.8) | | 76.4 (155.1) | | 38.4 (111.0) |
| Physiologist visits (mean, sd) | | | 7.1 (23.0) | | 11.3 (29.4) | | 7.7 (24.1) |
| Hospital admissions (mean, sd) | | | 4.5 (7.4) | | 7.0 (14.3) | | 4.9 (8.8) |

Additional file 5. Risk factors for logistic regression model

| **Variable** | **CV complication** | | **Other complication** | | **All-cause mortality** | |
| --- | --- | --- | --- | --- | --- | --- |
| **Gender (F vs M)** | 0.84 (0.76; 0.92) |  | 0.70 (0.59; 0.82) |  | 0.83 (0.75; 0.91) |  |
| **Age class (ref 35-54)** |  |  |  |  |  |  |
| 55-64 | 1.33 (1.02; 1.72) |  | 1.23 (1.03, 1.46) |  | 1.69 (1.25; 2.29) |  |
| 65-74 | 1.66 (1.29; 2.13) |  | 1.39 (1.17, 1.65) |  | 2.38 (1.78; 3.17) |  |
| >74 | 2.77 (2.16; 3.56) |  | 2.07 (1.75, 2.44) |  | 6.16 (4.65; 8.17) |  |
| **CCI class (Ref = 1)**  2 | **-** |  | **-** |  | 0.98 (0.82; 1.17) |  |
| 3 | - |  | - |  | 1.27 (1.07; 1.51) |  |
| ≥4 | - |  | - |  | 1.69 (1.32; 2.16) |  |
| **aDCSI class (Ref = 0)**  1 | 1.20 (1.03; 1.4) |  | 1.57 (1.21; 2.03) |  | 1.09 (0.93; 1.28) |  |
| 2 | 1.17 (1.00; 1.37) |  | 1.54 (1.18; 2.02) |  | 1.60 (1.39; 1.85) |  |
| 3 | 1.39 (1.11; 1.76) |  | 1.36 (0.88; 2.10) |  | 1.61 (1.28; 2.03) |  |
| 4 | 1.31 (1.03; 1.67) |  | 1.92 (1.29; 2.84) |  | 2.17 (1.75; 2.70) |  |
| 5 | 1.21 (0.98; 1.50) |  | 1.34 (0.91; 1.97) |  | 2.93 (2.46; 3.49) |  |
| **LTD duration (years)** | 1.01 (1.00; 1.02) |  | 1.02 (1.01, 1.02) |  | 1.02 (1.01, 1.02) |  |
| **Antidiabetic treatment (Ref none)** |  |  |  |  |  |  |
| Insulin only | 1.24 (0.99; 1.54) |  | 1.26 (0.90; 1.76) |  | 1.11 (0.94; 1.31) |  |
| Insulin + antidiabetic | 1.15 (0.93; 1.42) |  | 0.96 (0.69; 1.33) |  | 0.75 (0.64; 0.88) |  |
| Monotherapy | 0.72 (0.59; 0.89) |  | 0.40 (0.29; 0.56) |  | 0.47 (0.40; 0.54) |  |
| Bitherapy | 0.83 (0.68; 1.02) |  | 0.48 (0.35; 0.67) |  | 0.45 (0.38; 0.53) |  |
| Tri-therapy | 0.92 (0.70; 1.20) |  | 0.53 (0.34; 0.84) |  | 0.45 (0.35; 0.58) |  |
| **Comorbidity** |  |  |  |  |  |  |
| Chronic CV disease | 1.78 (1.58; 2.00) |  | 1.81 (1.58, 2.08) |  | 1.80 (1.56; 2.08) |  |
| Psychiatric | 1.19 (1.07; 1.31) |  | 1.33 (1.21, 1.47) |  | 1.59 (1.35; 1.88) |  |
| Chronic end-stage renal disease | 2.13 (1.46, 3.09) |  | 2.34 (1.77, 3.10) |  | 1.70 (1.27, 2.27) |  |
| COPD | - |  | 1.15 (1.01, 1.29) |  | 1.76 (1.56; 2.00) |  |
| Liver or pancreas disease | - |  | 1.33 (1.09, 1.63) |  | 2.17 (1.81; 2.61) |  |
| Cancer | - |  | - |  | 2.62 (2.35, 2.92) |  |
| Neurodegenerative | - |  | - |  | 2.68 (2.31; 3.10) |  |
| Other LTD | - |  | - |  | 1.29 (1.10; 1.50) |  |
|  |  |  |  |  |  |  |
|  |  |  |  |  |  |  |
|  |  |  |  |  |  |  |
|  |  |  |  |  |  |  |

Note: CCI: Charlson Comorbidity Index; COPD, Chronic Obstructive Pulmonary Disease; CV, Cardiovascular; DSCI, Diabetes Severity and Comorbidity Index; LTD, Long Term Disease allowance.


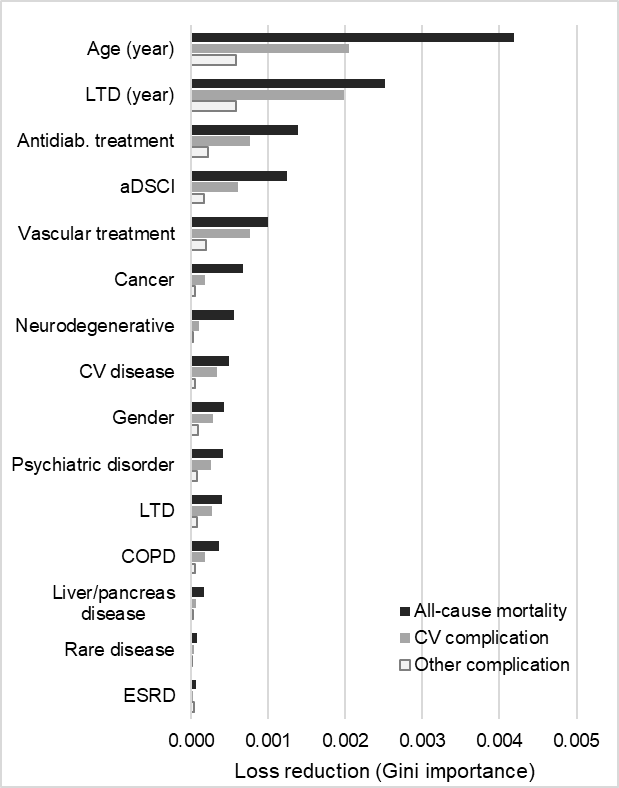


Additional file 6. Risk factors importance for Random Forest (RF) model

Note: COPD, Chronic Obstructive Pulmonary Disease; CV, Cardiovascular; aDSCI, Diabetes Severity and Comorbidity Index; ESRD: End stage of renal disease; LTD, Long Term Disease coverage.

Additional file 7.1 Calibration of models with observed versus median predicted risk for sub-groups patients with T2D

|  |  | **All patients** | **No event** | **With event** | **No CVD** | **With CVD** | **35-54y** | **55-64y** | **65-74y** | **75+** | **No trt** | **Insulin only** | **Insulin + antidiab.** | **Bith** | **Mono** | **Tri** |
| --- | --- | --- | --- | --- | --- | --- | --- | --- | --- | --- | --- | --- | --- | --- | --- | --- |
| **CV complications** | |  |  |  |  |  |  |  |  |  |  |  |  |  |  |  |
| Observed |  | 3.00% | - | - | 1.93% | 5.87% | 1.20% | 1.73% | 2.66% | 5.16% | 3.53% | 6.23% | 4.45% | 2.21% | 2.62% | 2.66% |
| Predicted | LR | 2.01% | 0.60% | 4.33% | 1.47% | 5.20% | 0.76% | 1.24% | 1.89% | 4.19% | 2.60% | 4.85% | 3.43% | 1.68% | 1.58% | 1.92% |
|  | RF | 1.82% | 0.42% | 4.84% | 1.22% | 5.34% | 0.68% | 1.16% | 1.83% | 4.13% | 2.41% | 4.66% | 3.24% | 1.49% | 1.39% | 1.73% |
|  | NN | 2.16% | 0.75% | 3.27% | 1.58% | 5.53% | 0.60% | 1.08% | 2.20% | 4.50% | 2.75% | 5.00% | 3.58% | 1.83% | 1.73% | 2.07% |
| **All complications** | |  |  |  |  |  |  |  |  |  |  |  |  |  |  |  |
| Observed |  | 1.00% | - | - | 0.75% | 1.52% | 0.69% | 0.72% | 0.86% | 1.37% | 1.12% | 2.57% | 1.77% | 0.74% | 0.52% | 0.74% |
| Predicted | LR | 0.63% | 0.60% | 1.27% | 0.46% | 1.24% | 0.42% | 0.52% | 0.64% | 0.82% | 1.18% | 2.14% | 1.41% | 0.51% | 0.39% | 0.61% |
|  | RF | 0.47% | 0.35% | 1.82% | 0.24% | 1.65% | 0.24% | 0.34% | 0.65% | 0.83% | 1.02% | 1.98% | 1.25% | 0.35% | 0.23% | 0.45% |
|  | NN | 0.59% | 0.60% | 0.19% | 0.40% | 1.90% | 0.23% | 0.33% | 1.06% | 1.24% | 1.14% | 2.10% | 1.37% | 0.47% | 0.35% | 0.57% |
| **All-cause mortality** | |  |  |  |  |  |  |  |  |  |  |  |  |  |  |  |
| Observed |  | 4.00% | - | - | 2.37% | 7.86% | 0.83% | 1.39% | 2.57% | 8.45% | 10.34% | 10.85% | 5.60% | 2.13% | 2.88% | 1.85% |
| Predicted | LR | 1.70% | 1.65% | 7.43% | 1.16% | 4.63% | 0.45% | 0.91% | 1.49% | 5.14% | 4.90% | 7.41% | 2.97% | 1.08% | 1.52% | 1.13% |
|  | RF | 1.42% | 1.39% | 9.56% | 1.02% | 4.31% | 0.47% | 0.93% | 0.82% | 4.47% | 4.62% | 7.13% | 2.69% | 0.80% | 1.24% | 0.85% |
|  | NN | 1.46% | 1.47% | 1.61% | 1.04% | 4.11% | 0.22% | 0.68% | 1.65% | 5.30% | 4.66% | 7.17% | 2.73% | 0.84% | 1.28% | 0.89% |

Note: Bith: Bitherapy; CV, Cardiovascular; LR, Logistic Regression; Mono: Monotherapy; NN, Neural Network; RF, Random Forest; RR: Relative Risk; Tri: Thritherapy.

| **A/ Acute CV complications**  **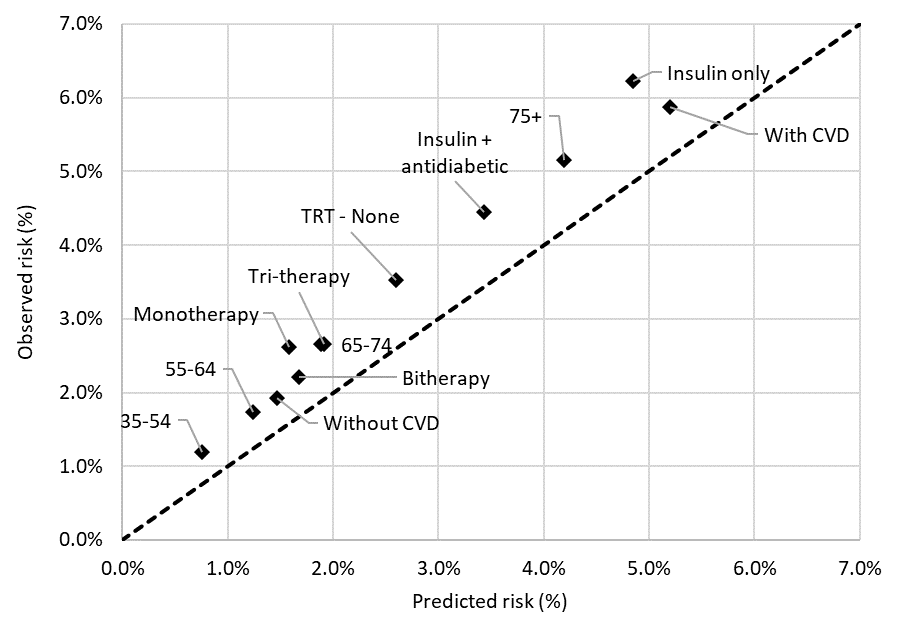** | **B/ Other acute complications**  **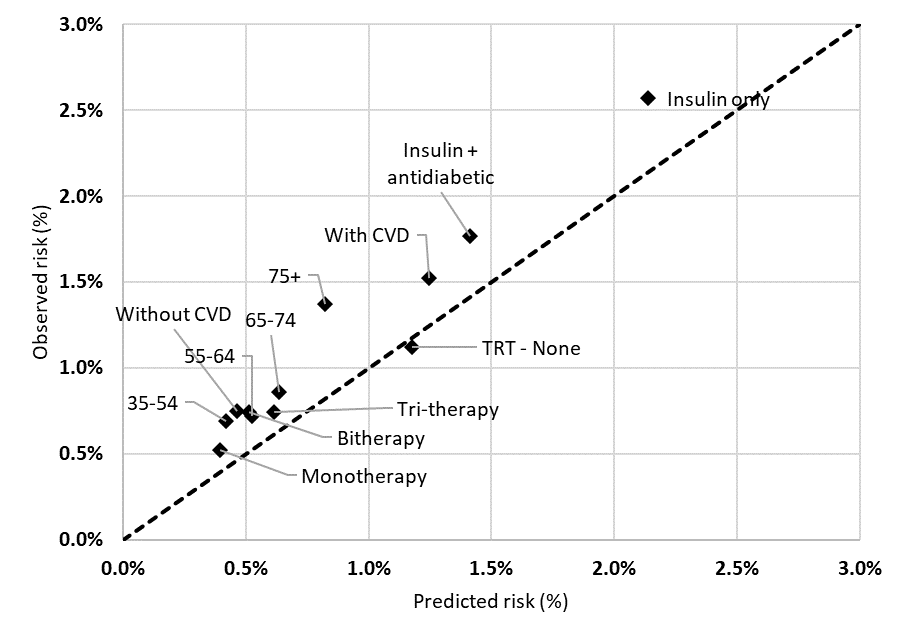** |
| --- | --- |
| **C/ All-cause mortality**  **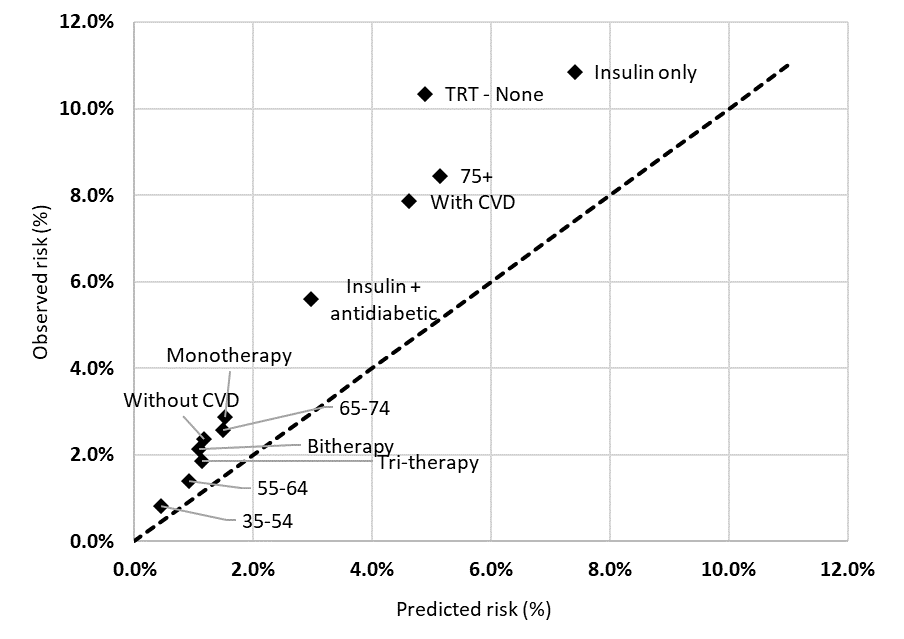** | **Additional file 7.2** **Observed versus median predicted risk estimated by RF model for sub-groups of patients with T2D**  The red 45° line represents a perfect prediction. |

| **A/ CV acute complication**  **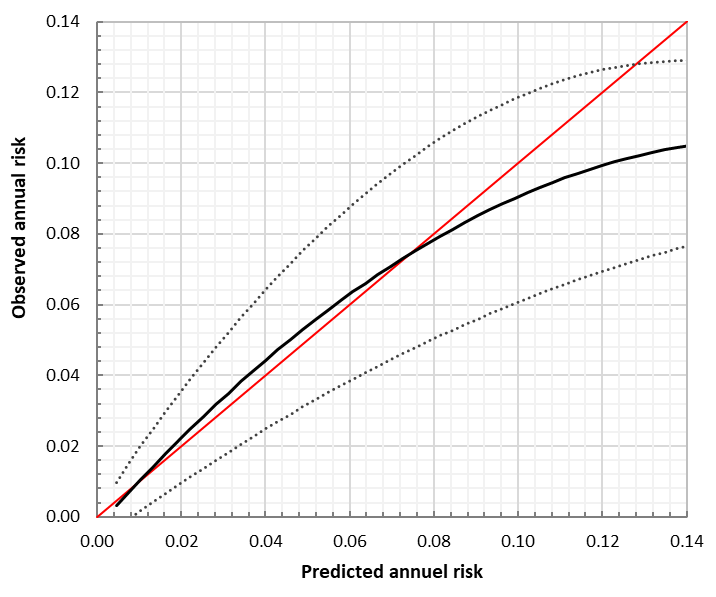** | **B/ Other acute complication**  **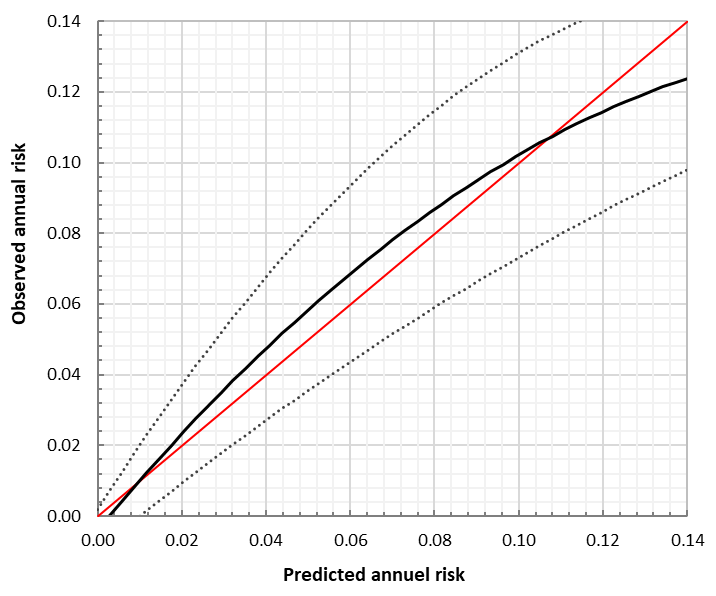** |
| --- | --- |
| **C All-cause mortality**  **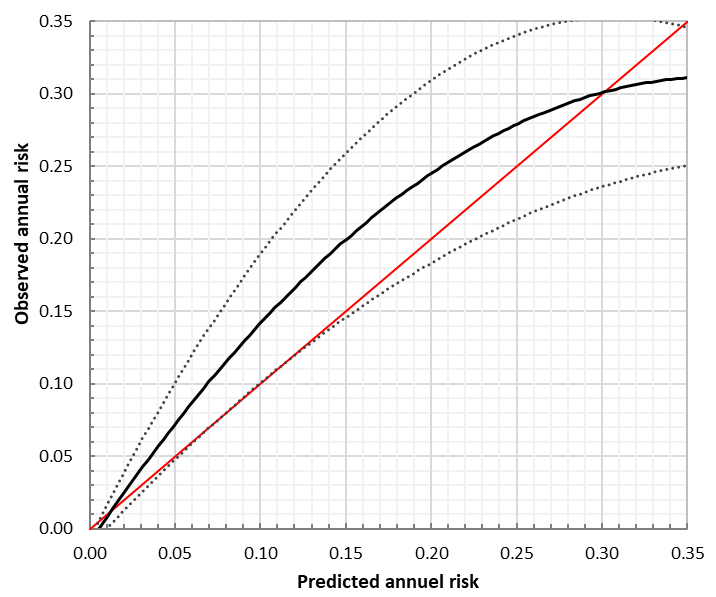** | **Additional file 7.3** **Calibration confidence interval for the goodness-of-fit between observed and predicted risk.**  The calibration interval shows the 95% CIs for observed outcome at predicted levels of risk. The red 45° line represents a perfect prediction. |
